# Supplementary material for: Foliar Selenate and Zinc Oxide Separately Applied to Two Pea Varieties: Effects on Growth Parameters and Accumulation of Minerals and Macronutrients in Seeds under Field Conditions
Source: Foods. 2023 Mar 17;12(6):1286. doi: 10.3390/foods12061286 (PMC10048356; doi:10.3390/foods12061286)
Supplement: Supplementary file 1 [file foods-12-01286-s001.zip › foods-2250412-supplementary.pdf]

Online Supplementary Information

Table S1. Correlation analysis between Se/Zn application dose and seed Se/Zn concentration in the studied pea varieties.

Treatment	Variety	Regression Equation	r^2	*p*-Value
Se				
	Ambassador	y = 0.0385x + 0.0907	0.999	0.004
	Premium	y = 0.0318x + 0.0638	0.999	0.002
Zn				
	Ambassador	y = −0.0093x + 43.953	0.999	0.019
	Premium	y = 0.0057x + 32.016	0.722	0.354
